# Serum uric acid and diabetic nephropathy

**DOI:** 10.12861/jrip.2012.13

**Published:** 2012-01-01

**Authors:** Ali Momeni

**Affiliations:** ^1^Department of Nephrology, Shahrekord University of Medical Sciences, Shahrekord, Iran

**Keywords:** Diabetic nephropathy, Uric acid, End-stage renal disease

**Implication for health policy/practice/research/medical:**

Diabetic nephropathy is the most common cause of end-stage renal disease worldwide. Recently, some prospective randomized controlled trials suggested that lowering of uric acid with allopurinol could decrease the severity of proteinuria and probably slow the progression of renal failure in diabetic patients. Mechanism of beneficial effect of xanthine oxidase inhibitor may related to preventing uric acid-induced renal inflammation.

Diabetic nephropathy is the most common cause of end stage renal disease worldwide. The pathogenesis of diabetic nephropathy is incompletely understood but may be include glycosylation of circulating and intrarenal proteins and abnormal intrarenal hemodynamics. Hyperglycemia may cause increase mesangial cell glucose concentration and glycation of matrix proteins that lead to increased matrix production and mesangial cell apoptosis (1). Cytokines activation, inflammation, and vascular growth factors may be responsible in the matrix accumulation in diabetic nephropathy (2).Glomerular hypertension and hyperfiltration are also responsible for development and progression of in diabetic nephropathy, hence, blockade of the renin-angiotensin system is beneficial in the treatment of disease. Detection of microalbuminuria is the screening method of choice for identifying of early stage of diabetic nephropathy (3). Correlation of serum uric acid (UA) with diabetic nephropathy was shown in several studies. For example level of serum UA was shown significantly higher in type 1 diabetic patients with persistent macroalbuminuria, compared to patients with normoalbuminuria (4). Elevated UA is associated with endothelial dysfunction, insulin resistance, development of hypertension, and cardiovascular disease (5). Elevated serum uric acid may be also associated with progression of non-diabetic renal disease (6). Currently renin-angiotensin system blockade is the gold standard in diabetic nephropathy treatment that lead to slowing the renal impairment but not arrest or reverse of the disease. Thus we require adjunctive therapeutic strategies, especially in patients with complications of treatment or lack of appropriate response. Recently, some prospective randomized controlled trials suggested that lowering of uric acid with allopurinol could decrease the severity of proteinuria and probably slow the progression of renal failure in diabetic patients (7) and also in the patients with hyperuricemia and non-diabetic chronic kidney disease (8). Mechanism of beneficial effect of xanthine oxidase inhibitor may related to preventing uric acid-induced renal inflammation (9). Indeed, allopurinol decrease serum uric acid level and reduce oxidative stress, it is not exactly obvious the main beneficial mechanism of allopurinol in the diabetic nephropathy. In conclusion, it seems that further studies are needed to clarify the effect of uric acid in initiation and progression of diabetic nephropathy and effect of uric acid lowering drugs on preventing or slowing of disease progression.

## Authors’ contributions

AM is the single author of the manuscript.

## Conflict of interests

The author declared no competing interests.

## Ethical considerations

Ethical issues (including plagiarism, informed consent, misconduct, double publication and redundancy) have been completely observed by the authors.

## Funding/Support

None declared
